# Characteristics of PM_2.5_ Chemical Compositions and Their Effect on Atmospheric Visibility in Urban Beijing, China during the Heating Season

**DOI:** 10.3390/ijerph15091924

**Published:** 2018-09-04

**Authors:** Xing Li, Shanshan Li, Qiulin Xiong, Xingchuan Yang, Mengxi Qi, Wenji Zhao, Xinlong Wang

**Affiliations:** 1 College of Resources, Environment & Tourism, Capital Normal University, Beijing 100048, China; lix2251575807@163.com (X.L.); mxoyxc@163.com (X.Y.); Qmengxi21@163.com (M.Q.); woaiwangxinlong88@163.com (X.W.); 2 Environmental Technology Consultancy, Beijing Municipal Research Institute of Environmental Protection, Beijing 100037, China; liss0502@163.com; 3 Faculty of Geomatics, East China University of Technology, Nanchang 330013, China; xiong_ql@163.com

**Keywords:** PM_2.5_, chemical compositions, pollution characteristics, extinction contribution

## Abstract

Beijing, which is the capital of China, suffers from severe Fine Particles (PM_2.5_) pollution during the heating season. In order to take measures to control the PM_2.5_ pollution and improve the atmospheric environmental quality, daily PM_2.5_ samples were collected at an urban site from 15 November to 31 December 2016, characteristics of PM_2.5_ chemical compositions and their effect on atmospheric visibility were analyzed. It was found that the daily average mass concentrations of PM_2.5_ ranged from 7.64 to 383.00 μg m^−3^, with an average concentration of 114.17 μg m^−3^. On average, the Organic Carbon (OC) and Elemental Carbon (EC) contributed 21.39% and 5.21% to PM_2.5_, respectively. Secondary inorganic ions (SNA: SO_4_^2^^−^ + NO_3_^−^ + NH_4_^+^) dominated the Water-Soluble Inorganic Ions (WSIIs) and they accounted for 47.09% of PM_2.5_. The mass concentrations of NH_4_^+^, NO_3_^−^ and SO_4_^2−^ during the highly polluted period were 8.08, 8.88 and 6.85 times greater, respectively, than during the clean period, which contributed most to the serious PM_2.5_ pollution through the secondary transformation of NO_2_, SO_2_ and NH_3_. During the highly polluted period, NH_4_NO_3_ contributed most to the reconstruction extinction coefficient (*b′_ext_*), accounting for 35.7%, followed by (NH_4_)_2_SO_4_ (34.44%) and Organic Matter (OM: 15.24%). The acidity of PM_2.5_ in Beijing was weakly acid. Acidity of PM_2.5_ and relatively high humidity could aggravate PM_2.5_ pollution and visibility impairment by promoting the generation of secondary aerosol. Local motor vehicles contributed the most to NO_3_^−^, OC, and visibility impairment in urban Beijing. Other sources of pollution in the area surrounding urban Beijing, including coal burning, agricultural sources, and industrial sources in the Hebei, Shandong, and Henan provinces, released large amounts of SO_2_, NH_3_, and NO_2_. These, which were transformed into SO_4_^2−^, NH_4_^+^, and NO_3_^−^ during the transmission process, respectively, and had a great impact on atmospheric visibility impairment.

## 1. Introduction

In recent years, air pollution in China has shown regional characteristics, in particular, regional haze, which is characterized by PM_2.5_ has become the major environmental problem [1]. PM_2.5_ damage is not only the atmospheric visibility impairment, but it also effects climate change, and, most importantly, human health [2,3]. The PM_2.5_ pollution problem had been attracted the wide attention in China and worldwide also. Beijing is the political, economic, and cultural exchange center of China, with a population of more than 21 million and a vehicle fleet of approximately 5.7 million (Beijing Statistical Yearbook of 2017) [4]. It is located in the Beijing-Tianjin-Hebei (BTH) region, where the air quality is the worst in China, and 8 of the 10 heavily polluted cities in China located there. Beijing have been facing the serious and complex PM_2.5_ pollution due to the rapid economic growth, population expansion, and a sharp increase in motor vehicle numbers and regional transport [5,6]. Especially, the air quality during the heating season (A period of municipal collective heating in Beijing normally starts from 15 November and ends on 15 March) was usually the worst as compared to the rest of the year because of the enhanced consumption of fossil fuel and the high frequency of stagnant weather.

In the past, a significant amount of work on the chemical compositions of PM_2.5_ has been carried out to reveal the formation mechanism of PM_2.5_ in Beijing. It was found that PM_2.5_ is a mixture of a variety of chemical species (e.g., Water-Soluble Inorganic Ions (WSIIs), Organic Carbon (OC), Elemental Carbon (EC), crustal, and trace elements) originated from natural and anthropogenic sources [7]. WSIIs were major components of PM_2.5_, accounting for more than 30.0% of PM_2.5_ [8]. The contributions of OC and EC to PM_2.5_ were about 15.0% and 5.0%, respectively [9]. Crustal elements, including calcium (Ca), iron (Fe), aluminum (Al), magnesium (Mg), etc., accounted for about 10% of PM_2.5_. While trace elements, including nickel (Ni), copper (Cu), zinc (Zn), selenium (Se), manganese (Mn), lead (Pb), and so on, only accounted for about 1% of PM_2.5_ [10]. Enrichment Factor (EF) analysis showed that trace elements mainly came from anthropogenic emissions and their enrichment in the fine fraction were more was elevated than in the coarse fraction [11]. Due to the continuous changes in source emissions, meteorology, photochemical reactions, planetary boundary layer heights, regional transport, etc. [12], the characteristics of PM_2.5_ chemical components and the driving factor for PM_2.5_ pollution showed temporally dynamic variations. For example, secondary inorganic ions (SNA) even accounted for more than 90% of the WSIIs during haze days in summer, the great enhancement of SNA concentration contributed most to PM_2.5_ pollution due to the secondary transformation under relatively high humidity [13]. In the winter, the average concentration of OC was higher than that of another season, because coal combustion for heating contributed more to PM_2.5_ pollution [9]. Besides, the proportion of elements in PM_2.5_ decreased with the increase of PM_2.5_ pollution, the enrichment of elements was not the main reason for the PM_2.5_ pollution [14].

In summary, previous studies have provided a scientific basis for the control of PM_2.5_ pollution in Beijing. However, most studies have focused on the characteristics of only one or two chemical components (WSIIs, carbon component, or element) of PM_2.5_ for a typical pollution event or seasonal variation. Few researchers have carried out systematically comprehensive studies on the characteristics of three PM_2.5_ chemical components and their effects on the extinction coefficient contribution under different levels of PM_2.5_ pollution during the heating season. For example, Tian et al. conducted analysis of characteristics of two chemical components (WSIIs, carbon component) in PM_2.5_ and PM_10_ and their effects on the scattering coefficient from 26 May to 30 June of 2012 at an urban site in Beijing [15]. Wu et al. investigated the relationship between visibility and the PM_2.5_ mass concentration, but they conducted no in-depth analysis of the contribution of PM_2.5_ chemical components to the extinction coefficient [16].

Therefore, in this study, daily PM_2.5_ sampling was conducted on the roof of a multifunction hall in Capital Normal University in Beijing from 15 November to 31 December 2016. The characteristics of three PM_2.5_ chemical components (including mass concentration, mass concentration ratios, oxidation ratios, aerosol acidity) and their effect on atmospheric visibility were analyzed in detail. Major chemical components that significantly contribute to PM_2.5_ pollution and atmospheric visibility impairment under different PM_2.5_ pollution levels were identified. The influence of factors, such as meteorological factors, sources, and regional transmission were examined by employing multiple techniques. The results of this study provide useful references for the increased control of PM_2.5_ pollution and sustainable urban management.

## 2. Materials and Methods

### 2.1. Aerosol Sampling

The PM_2.5_ sampling point was on the roof of multi-function hall (39°55′41″ N, 116°17′54″ E) of the Capital Normal University located in the Haidian district of Beijing, which is surrounded by residential areas, no major stationary air pollution sources were present within a circumference of 4 km of the site. Thus, the site was considered to be representative of a typical urban environment in Beijing.

The PM_2.5_ samples were collected for 24 h (from 8:00 a.m. to 8:00 a.m. of next day) by a large-flow PM_2.5_ sampler (TE-6070DV, Tisch, Medford, MA, USA) at a flow rate of 1.13 m^3^·min^−1^. The PM_2.5_ samples were collected on a 203 mm × 254 mm quartz filter paper (QMA, Whatman, UK). Before sampling, the quartz filters were baked at 500 °C for at least 4 h to remove adsorbed organic vapors. Filters were stored in a dryer for 24 h at constant temperature and humidity of 25 °C and 50%, respectively, before and after sampling. The airflow rate of the sampler was calibrated before and after the sampling to ensure that the instrument was working at the specified flow rate. According to the China’s Environmental Protection Standards (HJ633-2012) [17], we defined *ρ*(PM_2.5_) ≤ 75 μg m^−3^, 75 μg m^−3^ < *ρ*(PM_2.5_) ≤ 150 μg m^−3^, and 150 μg m^−3^ < *ρ*(PM_2.5_) as clean, lightly polluted, and highly polluted days, respectively. A total of 22 sets of PM_2.5_ samples were collected, including seven clean days, six lightly polluted days, and nine highly polluted days. 

Wind speed (WS), temperature (T), relative humidity (RH), atmospheric pressure, and sunshine duration were extracted from the China Meteorological Data Network (http://data.cma.cn/). The daily average PM_2.5_ mass concentration data for Haidian district were taken from the Beijing Environmental Protection Monitoring Center (http://www.china-jcw.cn/). Visibility data were taken from the weather station in the south of Beijing, the number of which is 54511.

### 2.2. Chemical Analysis

An area of 3 cm × 3 cm PM_2.5_ samples cuted from each quartz filter and blank filter were extracted in 45 mL of ultrapure water under ultrasonic agitation (18.2 MΩ·cm) for 30 min. Extracts were filtered through a syringe filter (pore size 0.45 µm) to remove the insoluble materials. The water-soluble anions (F^−^, NO_2_^−^, SO_4_^2−^, NO_3_^−^, and Cl^−^) and cations (NH_4_^+^, Ca^2+^, Na^+^, Mg^2+^, and K^+^) were measured by Ion Chromatograph ICS-1100 (Dionex, Sunnyvale, CA, USA) and Ion Chromatograph DX-80 (Dionex, Sunnyvale, CA, USA), respectively. Three times of blank filter standard deviation was taken as the detection limit. The detection limits of F^−^, NO_2_^−^, SO_4_^2−^, NO_3_^−^, Cl^−^, NH_4_^+^, Ca^2+^, Na^+^, Mg^2+^, and K^+^ were 0.003, 0.005, 0.001, 0.001, 0.001, 0.041, 0.001, 0.001, 0.001, and 0.001μg m^−3^, respectively. The standard curve was well linear because the fitting degree (*R*^2^) of the standard curve was 0.999. After every 10 samples, a random replicate check was performed and the relative standard deviation (RSD) of each ion was less than 5% for reproducibility test. The recovery rate of the experiment was analyzed by matrix addition, which showed that the recovery rate of water soluble ions was between 94% and 110%, and the coefficient variation of recovery was between 2.67% and 3.01%.

An area of 0.529 cm^2^ punched from each quartz filter was analyzed for EC and OC by Model 2001A thermal/optical carbon analyzer (American Desert Research Institute, Paradise, NV, USA). Each day, the instrument was baked for 30 min to remove residual carbon material before sample analysis, and then the system was run to guarantee a blank value lower than 0.5 µg cm^−2^. A CH_4_/CO_2_ standard gas was used for the calibration of the instrument before and after sample analysis. One sample for every ten was randomly selected for parallel analysis and two further samples were measured every week to ensure the accuracy of the experiment. Comparison with similar models in the American desert indicates that the experimental error of total carbon (TC; OC + EC) was <5% and the experimental error of OC and EC all were less than 10% [18].

An area of 0.529 cm^2^ punched from each quartz filter was measured for six soil elements (Al, Mg, Fe, Cu, Mn, and Ca) by Inductively Coupled Plasma-mass Spectrometry ICP-MS 7500ce (Agilent, Santa Clara, CA, USA) after microwave digestion with 5% nitric acid (6 mL), hydrogen peroxide (2 mL), and hydrofluoric acid (0.25 mL). In order to guarantee the stability of the instrument, each sample was measured three times, and the RSD of the internal standard element (45Sc, 73Ge, 115In, 209Bi) should be less than 3%, otherwise the sample should be remeasured. The concentration of internal standard solution (part #5183-4680, Aglient, Santa Clara, CA, USA) was 1 μg L^−1^. 0.1 g Geochemical Standard Reference Sample Soil (GBW07403GSS-3, China) were treated with the same pretreatment as the PM_2.5_ samples for quality control in every experiment, and the element recovery rate was between 86% and 112%. Blank (5% HNO_3_) was measured every 10 samples and the results showed that the concentrations of element in the blank were less than 1% of the average analyte concentration of the target element, which guarantees the accuracy of the experiment.

Three blank quartz filter (QMA, 203 mm × 254 mm) were measured and then subtracted from the loaded filters in the above mentioned analysis.

### 2.3. Enrichment Factor (EF)

The EF can be used to study the enrichment degree of element in atmospheric particulate and determine whether it arises from natural or artificial sources [19]. We selected Al as the reference element because Al is stable, immune to human interference, and is widespread in the crust. In addition, the mean of A-layer soil elements in Beijing was selected as the background value (China Environmental Monitoring Station 1990) to calculate the EF of PM_2.5_ element via Formula (1), and the specific classification of EF is shown in Table 1.

(1) $$\mathrm{EF}={\left(C_{x}/C_{\mathrm{Al}}\right)}_{{\mathrm{PM}}_{2.5}}/{\left(C_{x}/C_{\mathrm{Al}}\right)}_{\mathrm{soil}}$$

where EF is the enrichment factor of *x*, (*C_x_*/*C*_Al_)_PM2.5_ is the concentration ratio of *x* to Al in the PM_2.5_ samples, (*C_x_*/*C*_Al_)_soil_ is the concentration ratio of *x* to Al in A-layer soil in Beijing, respectively.

### 2.4. ISORROPIA-II

In atmospheric chemistry, strong acidity, ion-balanced acidity ([H^+^]_Total_), and in situ acidity ([H^+^]_Ins_) are the three parameters used to express aerosol acidity [20]. While the strong acidity is calculated from the laboratory results that were obtained from the aqueous extracts of particulate matter, [H^+^]_Total_ and [H^+^]_Ins_ are estimated from the measured ion concentrations in the liquid phase of aerosols [21]. As the estimation of [H^+^]_Ins_ includes prevailing meteorological factors, it is thus a better indicator of the actual particle acidity [20,22].

Thermodynamic equilibrium models, considering the environmental factorsis, are considered to be the most accurate method to calculate [H^+^]_Ins_ of atmospheric particles [23]. Thermodynamic equilibrium models ISORROPIA-II was designed with high computational efficiency to facilitate its incorporation in large scale models and has seen wide usage [23]. So, in this analysis, we selected ISORROPIA-II to calculate the [H^+^]_Ins_ of PM_2.5_. The model are considered: (a) “forward” mode calculations, in which inputs to the model include T, RH, and the total (gas + aerosol) concentrations of aerosol precursors in the air parcel, and, (b) “reverse” calculations, in which inputs to the model include T, RH, and the concentration of aerosol species [24]. Highly time-resolved measurements of aerosol composition are frequently conducted without the corresponding gas-phase aerosol precursor measurements (HCl, HNO_3_, NH_3_), under this condition, we calculated the [H^+^]_Ins_ predictions of PM_2.5_ while using “reverse” calculations. The aerosol state was set to solid+liquid and the following input parameters were used: T, RH, mass concentrations of NO_3_^−^, SO_4_^2−^, NH_4_^+^, Cl^−^, Na^+^, K^+^, Ca^2+^, and Mg^2+^ of PM_2.5_ in atmospheric.

### 2.5. TrajStat

TrajStat was developed by the Ya-Qiang Wang team of Centre for Atmosphere Watch and Chinese Academy of Meteorological Sciences. TrajStat is a free software with some trajectory statistics functions. The trajectory calculation module of HYSPLIT (http://www.arl.noaa.gov/ready/hysplit4.html) was included in TrajStat as an external process to calculate trajectories. The software also could do Potential Source Contribution Function (PSCF) and Concentration Weighted Trajectory (CWT) analysis, which is useful to identify the potential pollution sources spatially.

The PSCF values for the grid cells in the study domain are calculated by counting the trajectory segment endpoints that terminate within each cell [24]. The number of endpoints that fall in the *ij*th cell is designated *n_ij_*. The number of endpoints for the same cell having arrival times at the sampling site corresponding to PM concentrations higher than an arbitrarily set criterion is defined to be *m_ij_*. The PSCF value for the *ij*th cell is then defined as *m_ij_*/*n_ij_* value. The PSCF value can be interpreted as the conditional probability that the concentrations of a given analyte greater than the criterion level are related to the passage of air parcels through the *ij*th cell during transport to the receptor site. That is, cells high PSCF values are associated with the arrival of air parcels at the receptor site that have concentrations of the analyte higher than the criterion value. These cells are indicative of areas of ‘high potential’ contributions for the Constituent.

(2) $${\mathrm{PSCF}}_{ij}=\frac{m_{ij}}{n_{ij}}\cdot W(n_{ij})$$

To reduce the effect of small values of *n_ij_*, the PSCF values were multiplied by an arbitrary weight function *W_ij_* to better reflect the uncertainty in the values for these cells [25]. The weighting function reduced the PSCF values when the total number of the endpoints in a particular cell was less than about three times the average value of the end points per each cell. In this case, *W_ij_* was defined, as below.

(3) $$W(n_{ij})=\left\{ \begin{array}{l} 1.00,80\text{<}n_{ij}\\ 0.70,20\text{<}n_{ij}\leq 80\\ 0.42,10\text{<}n_{ij}\leq 20\\ 0.07,n_{ij}\leq 10 \end{array}\right.$$

A limitation of the PSCF method is that grid cells can have the same PSCF value when the sample concentrations are either only slightly higher or much higher than the criterion. As a result, it can be difficult to distinguish moderate sources from strong ones. In the CWT method [26], each grid cell is assigned a weighted concentration by averaging the sample concentration that have associated trajectories that crossed that grid cell, as follows:

(4) $$C_{ij}=\frac{1}{\sum_{l}^{M}\tau_{ijl}}\sum_{l=1}^{M}c_{l}\tau_{ijl}W(n_{ij})$$

where *C_ij_* is the average weighted concentration in the *ij*th cell, *l* is the index of the trajectory, *M* is the total number of trajectories, *C_l_* is the concentration observed on arrival of trajectory *l*, and τ*_ijl_* is the time spent in the *ij*th cell by trajectory *l*. A high value for *C_ij_* implies that air parcels traveling over the *ij*th cell would be, on average, associated with high concentration at the receptor. The arbitrary weighting function that is described above was also used in the CWT analyses to reduce the effect of the small values of *n_ij_*.

In this study, during the sampling period, the sampling point’s 72-h backward trajectories at a height of 500 m above ground level (AGL) and with a time interval of one hour were investigated. PSCF and CWT of PM_2.5_ were analyed with a spatial resolution of 0.5° × 0.5°, the set concentration criterion of PM_2.5_ was defined to be 75 μg m^−3^, according to the China’s Environmental Protection Standards (HJ633-2012).

### 2.6. Extinction Coefficient Reconstruction

It has been shown that the IMPROVE (IMPROVE (the Interagency Monitoring of Protected Visual Environments) method is successful in estimating the contribution of different chemical species to visibility decline in Beijing and other cities in China [15,27,28], therefore, the IMPROVE equation was used to reconstruct the extinction coefficient (*b′_ext_*) in this study. The contribution of CM (coarse particles) to *b_ext_* was ignored in this paper because it has been shown that the contribution of coarse particles to *b′_ext_* was very small [29]. The *b′_ext_* was therefore reconstructed according to the revised IMPROVE formula (Formula (5)).

(5) $$\begin{array}{l} {b\prime }_{ext}=2.2f_{s}(\mathrm{RH})\times \left[\mathrm{Small}({\mathrm{NH}}_{4}{)}_{2}{\mathrm{SO}}_{4}\right]+4.8f_{L}(\mathrm{RH})\times \left[{{\mathrm{Large}(\mathrm{NH}}_{4})}_{2}{\mathrm{SO}}_{4}\right]+2.4\times f_{s}(RH)\\ \times \left[{\mathrm{Small}\mathrm{NH}}_{4}{\mathrm{NO}}_{3}\right]+5.1\times f_{L}(RH)\times \left[{\mathrm{Large}\mathrm{NH}}_{4}{\mathrm{NO}}_{3}\right]+2.8\times \left[\mathrm{Small}\mathrm{OM}\right]+6.1\times \left[\mathrm{Large}\mathrm{OM}\right]\\ +1.7\times f_{ss}(\mathrm{RH})\times \left[\mathrm{SS}\right]+1\times \left[\mathrm{FS}\right]+0.6\left[\mathrm{CM}\right]+0.161\times \left[{\mathrm{NO}}_{2}\right]+10\left[\mathrm{EC}\right] \end{array}$$

where [(NH_4_)_2_SO_4_] = 1.375[SO_4_^2−^], [NH_4_NO_3_] = 1.29[NO_3_^−^], [SS] (Sea salt) = 1.8 × [Cl^−^], OM (organic matter) = 1. 6 × [OC], and [FS] (fine soil) = 2.2[Al] + 1.63[Ca] + 2.42[Fe] + 2.49[Si] + 1.94[Ti]. In this paper, the soil elements of Si and Ti were not detected synchronously, so only Al, Ca, and Fe were used to calculate FS. [Large *X*] = [Total *X*]^2^/(20 μg m^−3^) for [Total *X*] < 20 μg m^−3^ or [Large *X*] = [Total *X*] for [Total *X*] ≥ 20 μg m^−3^, [Small *X*] = [Total *X*] − [Large *X*]. The hygroscopic growth factor (*f*(RH)) of SO_4_^2−^, NO_3_^−^, and OM under specific humidity conditions was also considered, according to Pitchford et al. [30]. The unit of *b′_ext_* is Mm^−1^, and that of chemical compositions and NO_2_ all are μg m^−3^.

## 3. Results and Discussion

### 3.1. Mass Concentration of PM_2.5_

**Figure 1:**
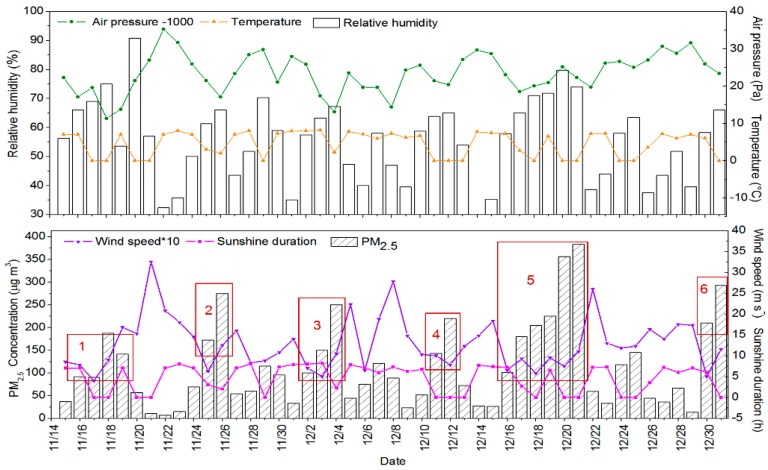
Daily variation of winter PM_2.5_ concentration and meteorological variables in Beijing, 2016.

During the sampling period, the daily average mass concentration of PM_2.5_ ranged from 7.64 to 383.00 μg m^−3^, with an average concentration of 114.17 μg m^−3^, which descended 35.13% during winter of 1997–2000 [31], but urban Beijing still suffered from serious PM_2.5_ pollution. As shown in Figure 1, 55.32% of days had the daily average mass concentration of PM_2.5_ exceeding the Grade II criterion of China’s daily Air Quality Standard (75 μg m^−3^), and 25.53% of days had the daily average mass concentration of PM_2.5_ exceeding 150 μg m^−3^. We assume that the daily average mass concentration of PM_2.5_ exceeding 75 μg m^−3^ and lasting for two days or even longer is a PM_2.5_ -polluted event, and a polluted event with one or more highly polluted days as a highly polluted event. On this basis, there was a total of six PM_2.5_ highly polluted events during the sampling period in Beijing, 2016.

During the six PM_2.5_ pollution events, the average mass concentration of PM_2.5_ was 198.44 μg m^−3^, representing an increase of 395.47% relative to clean days. Moreover, the average of relative humidity increased 44.10% relative to clean days, and the average of wind speed, sunshine duration, and atmospheric pressure decreased 46.09%, 44.82%, and 0.65%, respectively. This implies that the continuous stagnant weather with a high RH, a short sunshine duration, a low WS, and atmospheric pressure caused PM_2.5_ polluted events, especially the pollution event 5 with the longest polluted duration and the highest mass concentration of PM_2.5_.

### 3.2. Mass Concentration of Chemical Composition

#### 3.2.1. Inorganic Water-Soluble Ions

In this study, the average mass concentration of SNA was 53.76 μg m^−3^, which dominated the WSIIs and accounted for 47.09% of PM_2.5_

The average mass concentrations of the WSIIs under different PM_2.5_ pollution levels are summarized in Table 2. SNA accounted for 69.82% of the total WSIIs during the clean period, and then increased to 86.51% during the highly polluted period. The mass concentration of NH_4_^+^, NO_3_^−^, and SO_4_^2−^ during the highly polluted period were 8.08, 8.88, and 6.85 times greater, respectively, than during the clean period. Thus, the increase in NH_4_^+^, NO_3_^−^, and SO_4_^2−^ concentrations had a major impact on PM_2.5_ pollution.

The average concentration and contribution of SNA to PM_2.5_ during the sampling period in 2016 increased slightly than during winter in the years 1997–2000 (Table 3), which could be attributed to the increase in the number of motor vehicles. K^+^ was identified as a tracer for biomass burning [32], whose average mass concentration decreased significantly (Table 3), owing to the strict control over biological combustion in Beijing and surrounding areas.

#### 3.2.2. Carbon Component

The TC consists of OC and EC, EC is produced by incomplete combustion, of the two components making up OC, POC is emitted directly from the burning source, whereas SOC is derived from POC by photochemical chemical reaction in the atmosphere. On average, OC and EC with a mass concentration of 24.42 and 5.95 μg m^−3^ contributed 21.39% and 5.21% to PM_2.5_, respectively.

Table 2 shows that the concentration of TC were 10.77, 32.73, and 44.05 μg m^−3^ during the clean, lightly polluted, and highly polluted periods, respectively. OC represented the main carbon component, accounting for 81.00%, 78.94%, and 81.15% of TC during the clean, lightly polluted, and highly polluted periods, respectively. The mass concentration of OC increased during the highly polluted period, at 4.12 times greater than those during the clean period. Therefore, the increase of OC also had an impact on the PM_2.5_ pollution.

At the end of the 20th century, coal accounted for approximately 70% of the annual energy consumption, while natural gas accounted for less than 2% (China statistical yearbook). Up till the end of 2016, the proportion of coal consumption in the energy structure decreased to 9.81%, while that natural gas and electricity increased to 30% and 23.86% (China statistical yearbook), respectively. Thus, the average mass concentrations of EC and OC during the sampling period in 2016 significantly decreased when compared with the concentrations that were measured during winter in the years 1997–2000 (Table 3), which should be related to the above mentioned energy restructuring.

#### 3.2.3. Element

TE contributed an average of 11.34% to PM_2.5_. The mass concentration of TE in PM_2.5_ changed less than the other substances under different PM_2.5_ pollution levels (Table 4), which indicates that the enrichment of TE was not the main cause of PM_2.5_ pollution. EF analysis showed that Fe and Al was not enriched under different PM_2.5_ pollution levels; hence, they mainly came from soil dust. On the other hand, both Ca and Mg showed slight enrichment, so they could be disturbed by construction dust [33]. During the polluted period, Cu and Mn showed high and slight enrichment, respectively, so they could have originated from industrial emissions, such as iron and steel manufacturing [34] Moreover, Cu might also be affected by road dust, brake pads, and the exhaust of motor vehicles [31,35,36].

During the sampling period in 2016, the mass concentrations of Al, Ca, and Mg increased compared with the concentrations during winter in the years 1997–2000 (Table 3). This could be attributed to the increase in road and construction dust with the development of urban construction and the increase in the number of motor vehicles. Moreover, the large increase in Cu might be related to the increase in the number of motor vehicles, while the marked decline of Mn and Fe might be due to the relocation of industrial enterprises in Beijing.

### 3.3. Mass Concentration Ratio Analysis

The ratio of NO_3_^−^/SO_4_^2−^ has been used to identify the contributions from the stationary (mainly including coal burning) or mobile sources (mainly including motor vehicle) of NO_3_^−^ and SO_4_^2−^. This method indicates that motor vehicle exhaust is the main source of NO_3_^−^ and SO_4_^2−^ if NO_3_^−^/SO_4_^2−^ > 1, otherwise NO_3_^−^ and SO_4_^2−^ can be expected to largely originate from coal burning [5]. The rate of NO_3_^−^/SO_4_^2−^ was 1.32 in this study, indicating that the motor vehicles have surpassed coal burning and become the important primary emission sources of NO_3_^−^ and SO_4_^2−^ in PM_2.5_.

**Figure 2:**
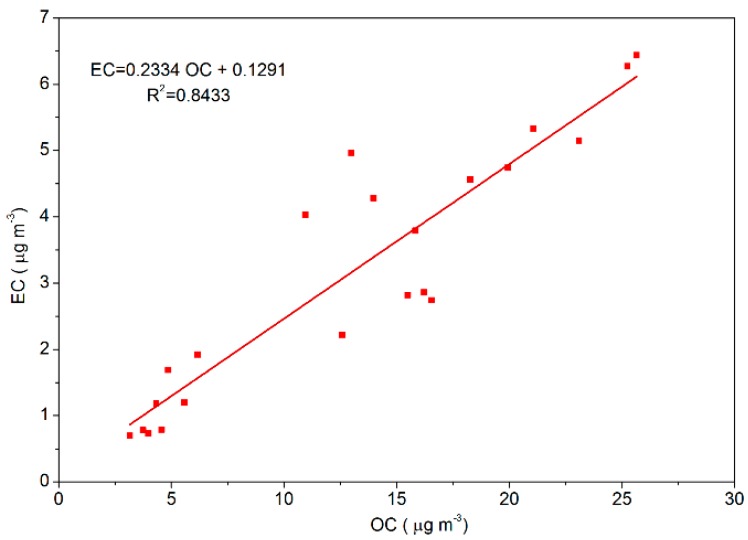
Correlation analysis between Elemental Carbon (EC) and Organic Carbon (OC) in Beijing during the heating season in 2016.

In this study, the sources of OC and EC were similar because their correlation (the fitting degree: *R*^2^ = 0.85; Figure 2) was strong [37]. So, when the value of OC/EC was in the range of 2.5–5.0, vehicle exhaust emission was considered as the main source of OC and EC in PM_2.5_, whereas when the value was in the range of 5.00–10.50, coal combustion was the main source of OC and EC [38,39]. The ratio of OC/EC in this study were 4.46, 3.90, and 4.50 during the clean, lightly polluted, and highly polluted periods, respectively, so we can know that vehicle exhaust emission was the main source of OC and EC in PM_2.5_ during the heating season in 2016.

### 3.4. Secondary Transformation

The SOR (Formula (6)) and NOR (Formula (7)) are good indicators of secondary transformation. It was reported that in the primary source emissions of SO_4_^2−^ and NO_3_^−^, SOR and NOR were smaller than 0.10, whereas SO_4_^2−^ and NO_3_^−^ were mainly produced by the secondary transformation of SO_2_ and NO_2_, and the higher values of SOR and NOR indicated that more gaseous species were oxidized to secondary aerosol in the atmosphere [40]. So, from Table 2, we can know that SO_4_^2−^ and NO_3_^−^ were mainly produced by the secondary transformation during polluted days.

SOR = nSO_4_^2−^/(nSO_4_^2−^ + nSO_2_) (6)

NOR = nNO_3_^−^/(nNO_3_^−^ + nNO_2_) (7)

where n refers to the molar concentration.

Many researchers have used the OC/EC minimum ratio method to estimate the size of POC and SOC, as shown in Formulas (8) and (9) [9,37]. The mass concentrations of POC and SOC under different pollution levels of PM_2.5_ are summarized in Table 2. SOC accounted for 40.85%, 32.88%, and 41.47% of OC during the clean, lightly polluted, and highly polluted periods, respectively. Therefore, SOC was an important ingredient of OC and widespread in the heating season.

**Figure 3:**
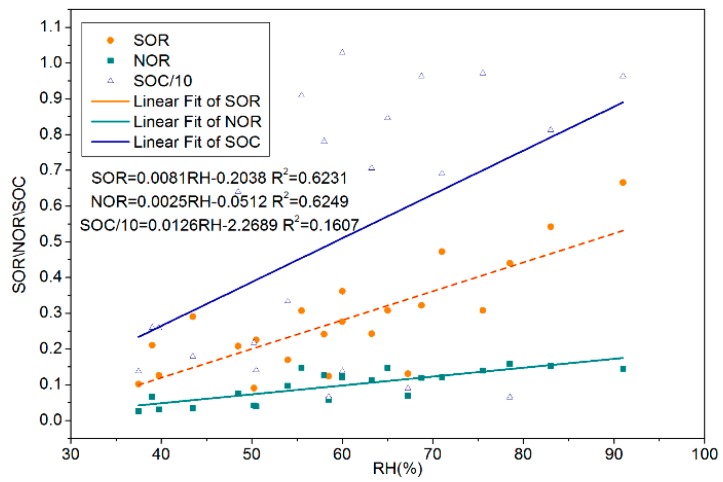
Linear regression analysis between Sulfur Oxidation Rate (SOR), Nitrogen Oxidation Rate (NOR), Secondary Organic Carbon (SOC), and Relative Humidity (RH) during the heating season in 2016.

Table 5 shows that both SOR and NOR have a significant positive correlation with relative humidity and do not have a significant correlation with WS, T, atmospheric pressure, and sunshine duration. The linear fitting analysis shows that both SOR and NOR had obvious linear relationship with RH, and the slopes of SOR and HR (0.0081) were greater than that of NOR and HR (0.0025), while no obvious linear relationship existed between SOC and RH (Figure 3), because the fitting degree (*R*^2^ = 0.1607) was too low. Thus, HR could promote the generation of SO_4_^2−^ and NO_3_^−^, especially SO_4_^2−^, and it had little effect on the generation of SOC. VOCs and NO_X_ from motor vehicle exhaust were the major precursor of SOC and NO_3_^−^ and were important catalysts for increased atmospheric oxidation, which could aggravate the PM_2.5_ pollution [41].

(8) $$\mathrm{POC}=\mathrm{EC}\times {\left(\mathrm{OC}/\mathrm{EC}\right)}_{\min}$$

(9) SOC=OC−POC

where (OC/EC)_min_ is the minimum value of OC/EC during the sampling period.

### 3.5. [H^+^]_Ins_ of PM_2.5_

The [H^+^]_Ins_ of PM_2.5_ was calculated by the thermodynamic equilibrium models ISORROPIA-II. Table 2 shows that PM_2.5_ was weakly acidic in Beijing during the heating season in 2016, and the acidity of PM_2.5_ during the polluted days was stronger than that during clean days, this may be because more acidic pollutants (SO_4_^2−^, NO_3_^−^, etc.) were generated and alkaline substances (NH_4_^+^, etc.) could not neutralize them by acid-base neutralization reaction during polluted days. Acidic particles will not only cause damage to ocean biogeochemistry [42], post a threat to human health [43], but also promote the formation of secondary aerosols [44,45]. Therefore, controlling the acidity of aerosol by reducing acidic pollutants (SO_4_^2−^, NO_3_^−^, etc.) was very important to alleviate the PM_2.5_ pollution during the heating season.

### 3.6. Extinction Coefficient Contribution

**Figure 4:**
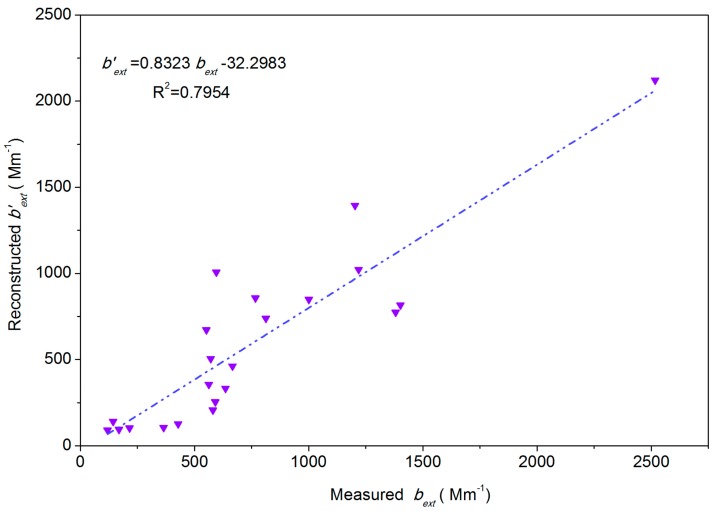
The reconstructed extinction coefficient (*b_ext_* ) and the measured extinction coefficient (*b′_ext_*) in Beijing during the sampling period in 2016.

**Figure 5:**
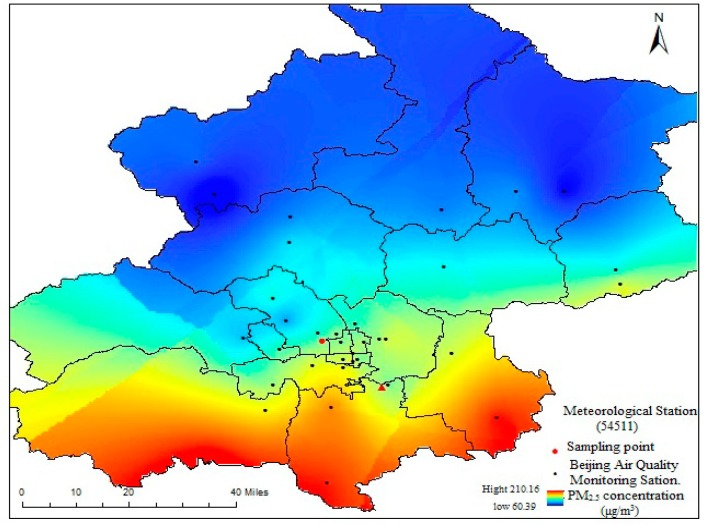
Spatial distribution of PM_2.5_ during the sampling period in 2016.

In this paper, visibility was measured every 1 h by Beijing Meteorological Station (54511), and the average daily actual-measured extinction coefficient (*b_ext_*) were the geometric mean of hourly *b_ext_* measurements per day [46]. The hourly *b_ext_* was calculated by Formula (10) [47]. Figure 4 indicates that daily *b′_ext_* were a little less than daily *b_ext_* in Beijing, December 2016, this may be because *b′_ext_* and *b_ext_* were affected by instantaneous visibility change, and, in addition, they were influenced by the spatial distribution of PM_2.5_ (Figure 5). Overall, *b′_ext_* can reflect the change trend of visibility in Beijing city during the heating season and there was a significant correlation (*R*^2^ = 0.81) between *b_ext_* and *b′_ext_*. So we can use the IMPROVE equation to calculate the extinction contribution of various chemical components of PM_2.5_ in Beijing during the heating season.

(10) $$b_{ext}=3.912\times 10^{6}/v$$

where the unit of visibility (*v*) and *b_ext_* is m and Mm^−1^, respectively.

**Figure 6:**
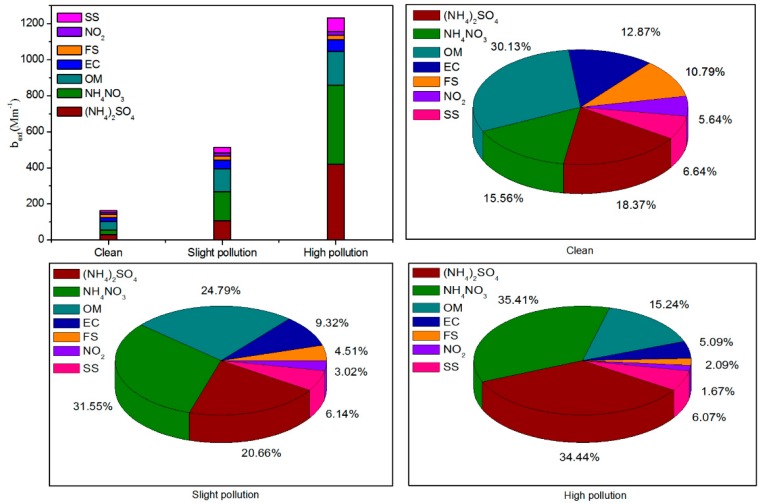
The *b′_ext_* and the contribution of PM_2.5_ chemical components to *b′_ext_* at different pollution levels of PM_2.5_ during the sampling period in 2016. Sea Salt (SS), Fine Soil (FS), Elemental Carbon (EC), Organic Matter (OM).

To estimate the contribution of different chemical species to the deterioration in visibility, *b′_ext_* was reconstructed for different pollution levels (clean, lightly polluted, and highly polluted) in Figure 6. During the clean, lightly polluted, and highly polluted periods, the average *b′_ext_* were 162.36, 513.22, and 1235.76 Mm^−1^, respectively. This result was consistent with the conclusion that the higher the PM_2.5_ mass concentration, the higher the *b′_ext_* value. During the clean period, OM contributed most to the *b′_ext_*, accounting for 30.13%, followed by NH_4_NO_3_ and (NH_4_)_2_SO_4_, accounting for 15.56% and 18.37%, respectively. With the increase of PM_2.5_ pollution level, the extinction contribution of NH_4_NO_3_ and (NH_4_)_2_SO_4_ gradually increased to 35.70% and 34.44%, respectively, during the highly polluted period, whereas the extinction contribution of OM gradually decreased to 18.37% (Figure 6). During the highly polluted period, NH_4_NO_3_ contributed the most to the decline in atmospheric visibility. This can be attributed to PM_2.5_ deliquescence under relatively high humidity, where a large amount of NO_2_, which is mainly from motor vehicle exhaust, were converted into NO_3_^−^ by liquid-phase oxidation reaction [41], and massive amounts of NH_3_ combined with NO_3_^−^ in PM_2.5_ by acid-base neutralization reaction. We also found that EC, FS, and SS contributed less to *b′_ext_* as compared with NH_4_NO_3_, (NH_4_)_2_SO_4_, and OM, but more than NO_2_ under different levels of PM_2.5_ pollution.

### 3.7. Regional Transport

**Figure 7:**
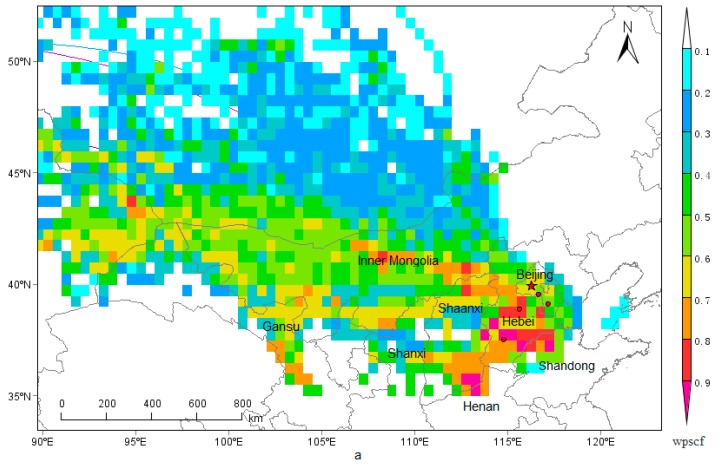

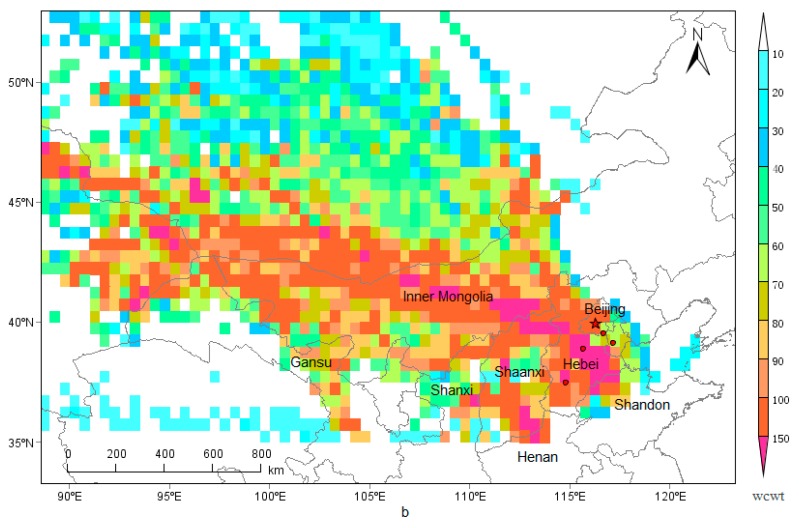
Potential Source Contribution Function (PSCF) (**a**) and Concentration Weighted Trajectory (CWT) (**b**) analysis based on the concentration of PM_2.5_.

The PSCF and CWT analysis of PM_2.5_ are displayed in Figure 7. Areas with high PSCF and CWT value of PM_2.5_ were mainly distributed in the central and southern Hebei, northwestern Shandong, northern Henan, central and western Inner Mongolia, eastern Gansu, northern Shanxi, and Shaanxi provinces, that is to say, the above mentioned areas were the potential emission source of PM_2.5_ in Beijng.

**Figure 8:**
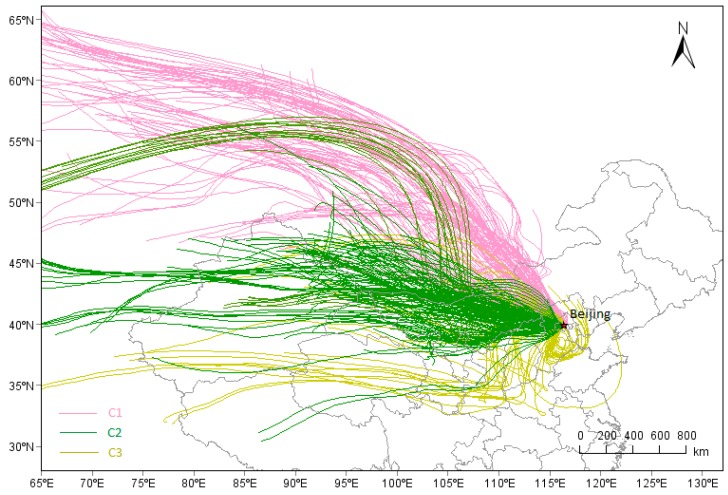
72-h air particle backward trajectory clusters during heating season in Beijing. Cluster 1 (C1), Cluster 2 (C2), Cluster 3 (C3).

In total, 528 transport trajectories of PM_2.5_ during samlping days were calculated, among which, three kinds of transport trajectories were clustered (Figure 8). PM_2.5_ and PM_2.5_-associated species of the three kinds of cluster (C1, C2, C3) are shown in Table 6. C1 represented the airflow from northwestern China, with higher concentration of Na^+^, Ca^2+^, Mg^2+^, Fe, and Al. The airflow with higher WS could carry considerable amounts of mineral aerosols from Inner Mongolia to Beijing, increasing the concentration of Na^+^, Ca^2+^, Mg^2+^, Fe, and Al.

C2 represented the middle-long distance transport from western China; the trajectories widely covered the Inner Mongolia, Gansu, Shanxi, and Shaanxi provinces, where a lot of coal were consumed for heating. The abundant OC and EC and were found in C2, indicating the possible burning of coal on the transmission path of C2.

C3 involved short distance mainly transport from northeastern Hebei provinces, covered the Shandong, Henan, Anhui, and Hebei Provinces. Both Shandong and Henan are provinces with large population and developed agriculture, southern Hebei Provinces are provinces with the highest consumption of coal, the most intensive heavy industry and the most serious polluted cities of China. These provinces could discharge abandent and complex pollutants (NH_3_, SO_2_, VOC, NO_X_, etc.), which could promote each other to hapen secondary transformation during the transmission, So, C3 displayed higher values of SNA, PM_2.5_ and *b′_ext_*. Higher concentration of Mn, Cu were found in C3, indicating industry-related components of the BTH region were carried to Beijing along with the airflow. The abundant K^+^ indicated the possible occurrence of biomass burning on the transmission path of C3.

## 4. Conclusions

During the heating season in 2016, urban Beijing suffered from severe PM_2.5_ pollution due to the high frequency of stagnant weather. The daily average mass concentration of PM_2.5_ ranged from 7.64 to 383.00 μg m^−3^, with an average concentration of 114.17 μg m^−3^. On average, SNA dominated the water-soluble ions, accounting for 47.09% of PM_2.5_, followed by OC (21.39%) and TE (11.34%). The mass concentrations of NH_4_^+^, NO_3_^−^, and SO_4_^2−^ during the highly polluted period were 8.08, 8.88, and 6.85 times greater, respectively, than during the clean period; these components were the biggest contributors to PM_2.5_ pollution. During the polluted period, both SOR and NOR were greater than 0.10; thus, SO_4_^2−^ and NO_3_^−^ were mainly produced by the secondary transformation of SO_2_ and NO_2_. The acidity of PM_2.5_ in Beijing was weakly acid. SOR and NOR had a significant positive correlation with relative humidity, but they did not have a significant correlation with WS, T, atmospheric pressure, and sunshine duration. Acidic PM_2.5_ and relatively high humidity promoted the generation of secondary aerosol and aggravated PM_2.5_ pollution. 

During the clean, lightly polluted, and highly polluted periods, the average *b′_ext_* values were 162.36, 513.22, and 1235.76 Mm^−1^, respectively. NO_3_^−^, SO_4_^2−^, NH_4_^+^, and OM were the major chemical components that reduced atmospheric visibility, and contributed over 70% of *b′_ext_* during the polluted period. This amount reached as high as 85.09% during the highly polluted period. NH_4_NO_3_ contributed the most to *b′_ext_*, accounting for 35.70%, followed by (NH_4_)_2_SO_4_, (34.44%), and OM (15.24%). EC, FS, and SS contributed less to *b′_ext_* when compared with NH_4_NO_3_, (NH_4_)_2_SO_4_, and OM. Local motor vehicles contributed the most to NO_3_^−^, OC, and visibility impairment in urban Beijing. Other sources of pollution in the area surrounding urban Beijing, including coal burning, agricultural sources, and industrial sources in the Hebei, Shandong, and Henan provinces, released large amounts of SO_2_, NH_3_, and NO_2_. These were transformed into SO_4_^2−^, NH_4_^+^, and NO_3_^−^ during the transmission process, respectively, and they had a great impact on atmospheric visibility impairment.

This study provides useful references for the further control of air quality and sustainable urban management during the heating season in Beijing. However, our study also has several limitations. For example, some conclusions may be different if the measurement period was longer than just two months of the same year. The chemical compositions of PM_2.5_ that were used in this study were analyzed by an offline method, which could raise questions about the validity of the results. Thus, in the future, longer measurement period combined with online analysis techniques are needed. 

## Figures and Tables

**Table 1:** Relationships between enrichment factor (EF) and enrichment degree of the studied elements.

EF	EF < 1	1 < EF < 10	10 < EF < 100	100 < EF < 1000	1000 < EF
Degree of enrichment	Almost no enrichment	Slight enrichment	Moderate enrichment	High enrichment	Super enrichment
Level	1	2	3	4	5
Source	Crustal or soil	Natural sources and human sources	Human sources	Human sources	Human sources

**Table 2:** PM_2.5_ chemical composition characteristics at different pollution levels of PM_2.5_.

Indicator	Clean	Lightly Polluted	Highly Polluted	Indicator	Clean	Lightly Polluted	Highly Polluted
WSIIs	15.24	62.07	94.76	OM	13.85	41.53	57.07
Na^+^	0.78	1.51	1.04	SOC	2.62	5.15	7.48
NH_4_^+^	2.05	9.26	16.49	POC	3.72	8.91	10.90
K^+^	0.42	1.79	2.95	TE	11.41	13.06	13.52
Ca^2+^	0.95	1.09	1.23	Al	2.78	3.17	3.13
Mg^2+^	0.09	0.11	0.13	Fe	0.77	1.16	1.46
F^−^	0.05	0.11	0.09	Ca	5.45	5.7	5.8
Cl^−^	1.99	3.76	6.96	Mg	2.32	2.83	2.90
NO_2_^−^	0.32	0.33	0.38	Cu	0.06	0.15	0.16
NO_3_^−^	3.94	23.95	34.97	Mn	0.03	0.05	0.07
SO_4_^2−^	4.65	20.16	30.52	[H^+^]_Ins_	6.7	6.1	6.3
TC	10.77	32.73	44.05	SOR	0.19	0.21	0.43
OC	8.66	25.96	35.67	NOR	0.05	0.10	0.14
EC	2.11	6.77	8.38	RH (%)	45.57	57.21	72.89

Organic Carbon (OC), Elemental Carbon (EC), Total Carbon (TC: OC+EC), Organic Matter (OM), Particulate Organic Carbon (POC), Secondary Organic Carbon (SOC), Total Element (TE), Sulfur Oxidation Rate (SOR), and the Nitrogen Oxidation Rate (NOR). The mass concentration unit of PM_2.5_ chemical compositions all are μg m^−3^.

**Table 3:** Interannual variation of concentration of PM_2.5_ and its major chemical compositions.

Sampling Time	SO_4_^2−^	NO_3_^−^	NH_4_^+^	K^+^	Cl^−^	Ca^2+^	Mg^2+^	NO_3_^−^/SO_4_^2−^	EC	OC	Mn	Cu	Al	Fe	Ca	Mg	PM_2.5_
1997–2000 [37]	24.87	15.35	7.80	2.55	-	-	-	<1	11.08	31.49	0.10	0.05	0.76	1.17	1.05	0.19	176.00
2016	20.03	23.56	10.17	1.46	3.91	1.06	0.11	1.28	5.95	24.42	0.05	0.14	3.30	1.14	5.56	2.76	114.17

No substance in the reference article (-). The unit of PM_2.5_ chemical compositions all are μg m^−3^.

**Table 4:** Average enrichment factor (EF) of elements in PM_2.5_ at different PM_2.5_ pollution levels.

Composition	Clean				Lightly Polluted				Highly Polluted			
	Concentration (µg m^−3^)	EF	Level	Degree	Concentration (µg m^−3^)	EF	Level	Degree	Concentration (µg m^−3^)	EF	Level	Degree
Fe	0.77	0.51	1	No	1.16	0.76	1	No	1.46	0.95	1	No
Ca	5.45	4.16	2	Slight	5.50	4.35	2	Slight	5.85	4.46	2	Slight
Mg	2.32	3.99	2	Slight	2.83	4.87	2	Slight	2.90	4.99	2	Slight
Al	2.78	1	1	No	3.58	1	1	No	3.52	1	1	No
Cu	0.06	67.17	3	Moderate	0.15	142.14	4	high	0.16	146.45	4	high
Mn	0.03	1.36	2	Slight	0.05	2.03	2	Slight	0.07	2.62	2	Slight

**Table 5:** Pearson correlation analysis of NOR, SOR, and meteorological elements and precursors.

Indicator	WS	HR	T	Atmospheric Pressure	Sunshine Duration
SOR	−0.148	0.750 **	−0.150	−0.281	−0.434 *
NOR	−0.491	0.790 **	−0.032	−0.422	−0.284
SOC	−0.450	0.501 *	−0.497 *	−0.093	−0.019

* indicates a significant correlation at the 0.05 level (bilateral); ** indicates a significant correlation at the 0.01 level (bilateral). *n* = 22. Wind Speed (WS), Relative Humidity (HR), Temperature (T).

**Table 6:** The statistical results of PM_2.5_ and PM_2.5_-associated species of each kind of cluster.

Cluster	Na^+^	Ca^2+^	Cl^−^	K^+^	NO_3_^−^	SO_4_^2−^	NH_4_^+^	EC	OC	Al	Fe	Cu	Mn	SOR	NOR	[H^+^]_Ins_	PM_2.5_	WS	RH	*b′_ext_*
Unit	μg m^−3^																	(m s^−1^)	%	Mm^−1^
C1	1.25	1.27	3.06	0.78	8.68	9.15	4.05	3.51	15.44	3.48	0.77	0.11	0.04	0.26	0.07	6.5	88.67	1.79	53.67	329.75
C2	1.21	1.38	4.78	1.89	22.43	14.54	8.62	7.89	31.89	3.68	0.54	0.14	0.03	0.29	0.12	7.2	169.28	1.21	64.46	679.53
C3	0.87	1.14	6.04	2.04	23.19	17.66	6.95	5.20	23.18	3.13	0.34	0.33	0.05	0.33	0.13	4.1	180.50	1.46	66.56	876.29
